# BLoss-DDNet: bending loss and dual-task decoding network for overlapping cell nucleus segmentation of cervical clinical LBC images

**DOI:** 10.3389/frai.2025.1649452

**Published:** 2025-11-26

**Authors:** Guihua Yang, Ziran Chen, Peng Guo, Junchi Ma, Jinjie Huang, Cong Jin, Xiaona Yang, Kai Zhao, Yibo Wang, Qi Gao, Chengcheng Liu, Tianqi Wu, Yong Li, Yingwei Guo, Jie Zheng, Xiangran Cai, Yingjian Yang

**Affiliations:** 1 School of Mechanical and Electrical Engineering, Daqing Normal University, Daqing, Heilongjiang, China; 2 Department of Radiological Research and Development, Shenzhen Lanmage Medical Technology Co., Ltd, Shenzhen, Guangdong, China; 3 College of Medicine and Biological Information Engineering, Northeastern University, Shenyang, China; 4 School of Automation, Harbin University of Science and Technology, Harbin, Heilongjiang, China; 5 School of Computer, Xi'an Aeronautical University, Xi'an, Shanxi, China; 6 School of Information Science and Engineering, Zaozhuang University, Zaozhuang, Shandong, China; 7 Department of Medical Image Processing Algorithm, Research and Development Center of Smart Imaging Software, Neusoft Medical System Co., Ltd, Shenyang, Liaoning, China; 8 School of Life and Health Management, Shenyang City University, Shenyang, China; 9 School of Electrical and Information Engineering, Northeast Petroleum University, Daqing, China; 10 Medical Imaging Center, First Affiliated Hospital of Jinan University, Guangzhou, China

**Keywords:** cervical cancer, cervical clinical LBC images, cell nucleus segmentation, bending loss, dual decoding network, convolutional neural network

## Abstract

**Introduction:**

Cervical cancer has become one of the most malignant tumors that threatens women's health worldwide. Liquid-based cytology (LBC) examination has become the most common screening method for detecting cervical cancer early and preventing it. Currently, nuclear segmentation technology for cervical clinical LBC images based on convolutional neural networks has become a vital means of assisting in the diagnosis of cervical cancer. However, the existing nuclear segmentation techniques fail to segment the nuclei of severely overlapping nuclei in highly aggregated cell clusters, which will inevitably lead to the misdiagnosis of cervical cancer pathology.

**Methods:**

Therefore, a novel bending loss and dual-task decoding network (Bloss-DDNet) is proposed for overlapping cell nucleus segmentation of cervical clinical LBC images. First, the network architecture search method is introduced to search and optimize the architecture of the decoding module in the dual-task branch, determining the mask and boundary decoding modules (dual-task decoding modules) of the Bloss-DDNet. Second, two feature maps, separately generated from dual-task decoding branches composed of a shared encoder module and dual-task decoder modules, are fused to enhance the sensitivity to cell nucleus boundaries. Third, a bending loss is introduced to the loss function to focus on the curvature variation characteristics of the intersection of overlapping cell nucleus boundaries, thereby constraining the training process of the dual-task decoding branch and increasing the constraint on the cell nucleus boundary.

**Results:**

The results show that all evaluation metrics of the proposed Bloss-DDNet achieved the best performance on public datasets.

**Discussion:**

Therefore, the proposed Bloss-DDNet can effectively address the segmentation problem of overlapping cell clusters and nuclei in clinical LBC images, providing strong support for subsequent clinical auxiliary diagnosis of cervical cancer.

## 1 Introduction

Cervical cancer is one of the most common malignant tumors threatening global women's health, occupying the first place in the incidence rate and mortality rate of female reproductive system tumors, and seriously threatening the health of female comrades (Bray et al., 2020; Wu et al., 2024; Huang et al., 2021; Yang et al., 2022). The latest statistics, released by the Global Cancer Observatory 2022 (https://gco.iarc.fr/en), which are updated every three years, indicate that there were 662,301 new cases and 348,874 deaths from cervical cancer worldwide, including 150,659 new cases and 55,694 deaths in China. From this, cervical cancer has caused serious harm to female comrades. The leading cause of cervical cancer is human papillomavirus infection, especially persistent infection of high-risk HPV types. However, cervical cancer has a long and reversible precancerous stage, and early diagnosis and treatment are crucial for improving the survival rate of cervical cancer patients.

Clinically, liquid-based cytology (LBC) is a widely used method for cervical cancer screening (Rodríguez et al., 2024). It involves collecting cervical cell samples and examining them under a microscope to detect cancerous lesions with changes in internal structure and morphology, as well as noncancerous lesions such as pathogenic microbial infections and inflammation. However, due to factors such as cervical cell overlap, morphological diversity, and subjectivity in manual interpretation, the accuracy and efficiency of cervical clinical LBC are somewhat limited.

The automatic nucleus segmentation of cervical clinical LBC images is of great significance for automated cervical cancer screening, as it can significantly improve the accuracy and efficiency of diagnosis (Huang et al., 2021; Yang et al., 2022). However, traditional segmentation algorithms such as level set (Lu et al., 2015) and threshold segmentation (Phoulady et al., 2016) poorly deal with complex backgrounds and overlapping cells. In recent years, deep learning techniques, especially convolutional neural networks (CNNs) and Transformers, have made significant progress in medical image processing (Yang et al., 2021; Zeng et al., 2023; Zaman et al., 2024; Ghosal et al., 2025; Sikarwar et al., 2025; Khan et al., 2025; Balakrishnan and Sengar, 2024; Sengar et al., 2023). In most cases, the performance of cell nucleus segmentation in cervical clinical LBC images using deep learning networks is superior to that of traditional algorithms (Liu et al., 2018).

Specifically, U-Net and its improved network have been introduced as a backbone for cell segmentation in various cellular images due to their outstanding performance (Huang et al., 2021; Yang et al., 2022, 2021; Zeng et al., 2023; Zaman et al., 2024; Hussain et al., 2020; Zhou et al., 2018). Meanwhile, Gu et al. (2019) proposed the CE Net, which utilizes an enhanced network architecture with DAC and RMP blocks to extend the U-Net. Although the CE Net can handle overlapping cell nuclei effectively, it exhibits high computational complexity and encounters memory limitations when processing large-scale datasets. In addition, Qu et al. (2019) proposed Joint segmentation, which can simultaneously predict cell nucleus segmentation and better capture the overall structure of cells. However, when dealing with overlapping cell nuclei, the computational complexity of the Joint segmentation is high. Subsequently, Lal et al. (2021) proposed the NucleiSegNet, a network specifically designed for cell nucleus segmentation, which introduces new robust residual blocks and attention mechanisms to extract and learn deep features. Although the NucleiSegNet performs well in handling overlapping cell nuclei and accurately detecting nuclei of different shapes, there is still room for improvement in its performance when dealing with highly overlapping cell nuclei.

**Figure 1:**
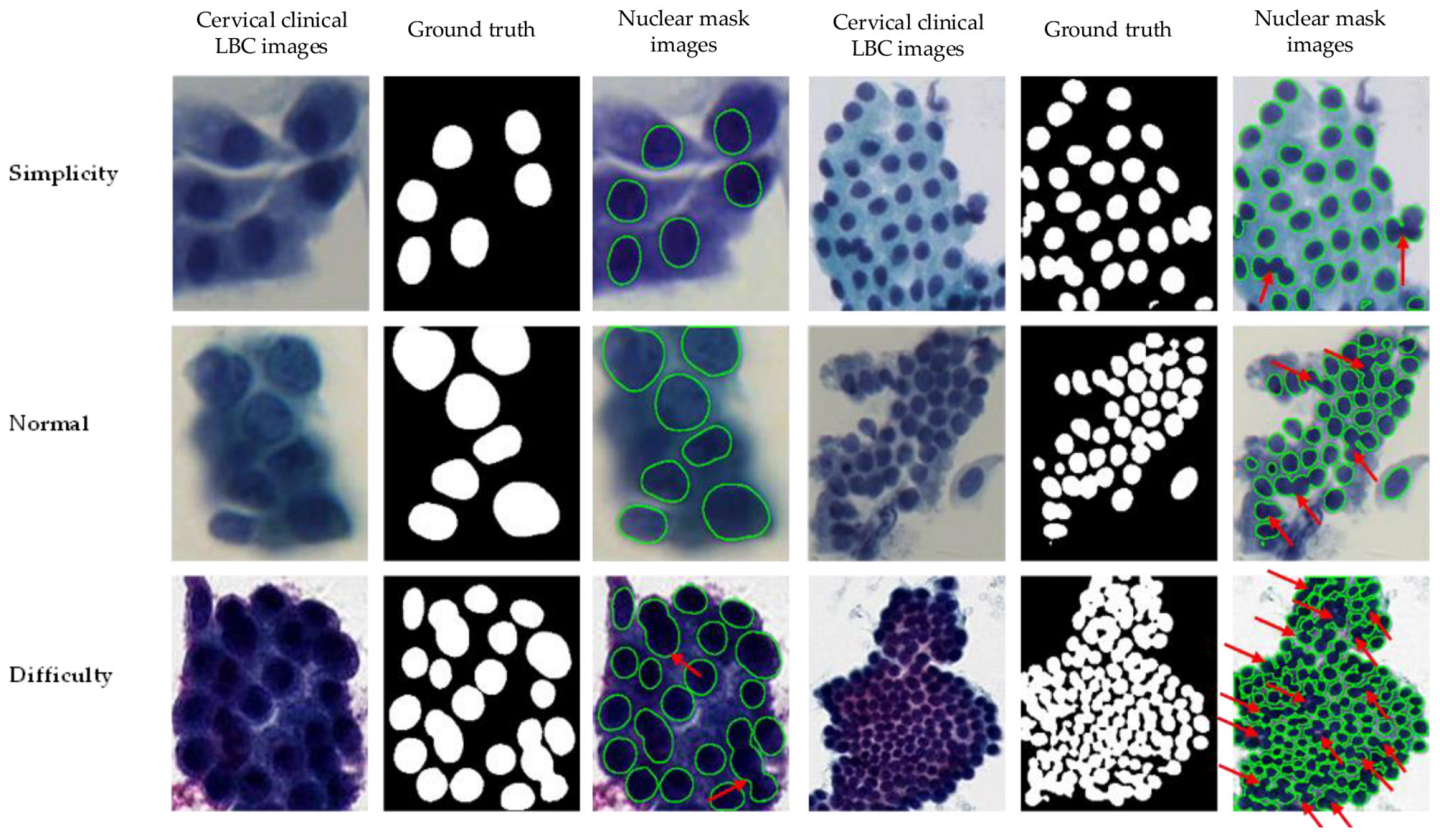
Typical nuclear boundary connectivity problem in cell nucleus segmentation of cervical clinical LBC images.

Furthermore, for cell nucleus segmentation of cervical clinical LBC images, Zhao et al. (2021) proposed that AL-net adjusts its network parameters through an adaptive learning mechanism to improve its adaptability to nuclei with varying degrees of overlap. Although the AL-net method has certain advantages in handling overlapping cell nuclei, its training process is relatively complex and requires a large amount of labeled data. In addition, we proposed the GCP-Net to improve the quality of feature learning through multi-scale context gating and global context attention (Yang et al., 2022). It has achieved a better performance than the above networks. Meanwhile, the fusion of CNNs and Transformers is a highly active and important research direction in the field of computer vision. The combination of the two can complement each other's strengths and weaknesses: CNN can efficiently extract local features of edges and textures, while the Transformer excels in modeling global contextual dependencies (Chen et al., 2024), such as those found in TransUNet (Chen et al., 2102). Specifically, TransUNet introduces a Transformer-centered encoder-decoder framework that combines self-attention and cross-attention in a sequence-to-sequence prediction context for medical image segmentation, thereby addressing the limitations of convolution-based methods, such as U-Net, in modeling long-range dependencies (Chen et al., 2102). In addition to TransUNet, the new semantic segmentation network, SegFormer, has attracted widespread attention due to its efficient, concise, and robust performance (Eskandari et al., 2023; Sharma et al., 2025). However, there is still a problem with connected nuclei, indicated by red arrows, in the cell segmentation results of cervical clinical LBC images shown in Figure 1. Therefore, it is necessary to propose a novel bending loss and dual-task decoding network (Bloss-DDNet) for overlapping cell nucleus segmentation of cervical clinical LBC images to eliminate the connectivity problem. Our contributions in this paper are briefly described as follows:

(1) Dual-task decoding branches composed of a shared encoder module and dual-task decoder modules are designed for decoding the mask and boundary of the overlapping cell nucleus segmentation of cervical clinical LBC images. In addition, two feature maps separately generated from dual-task decoding branches are fused to enhance the sensitivity of cell nucleus boundaries.

(2) The neural architecture search (NAS) framework is introduced for determining 1^#^ and 2^#^ decoder modules, obtaining the optimal combination and hyperparameter configuration of NAS-mask and NAS-boundary in the dual-task decoding branches.

(3) A bending loss, which focuses on the curvature variation characteristics of the intersection of overlapping cell nucleus boundaries, is introduced to the loss function to constrain the training process of the dual-task decoding branch and increase the constraint on the cell nucleus boundary.

(4) The proposed Bloss-DDNet can effectively solve the segmentation problem of overlapping cell clusters and nuclei in cervical clinical LBC images and may strongly support subsequent clinical auxiliary cervical cancer diagnosis.

## 2 Materials and methods

This paper proposes a BLoss-DDNet to solve the nuclear boundary connectivity problem in overlapping cell nucleus segmentation of cervical clinical LBC images. Based on the above, materials and methods are described in Sections 2.1 and 2.2, respectively.

### 2.1 Materials

Most existing public datasets of cervical clinical LBC images contain only a cervical cell in each image, making them unsuitable for the entire diagnostic process of real-world clinical case data. Therefore, this study selected CNSeg and MoNuSeg, which share overlapping cell nuclei, as the primary datasets among numerous publicly available datasets (Zhao et al., 2023). Subsequently, two Datasets (Dataset 1 and Dataset 2) are generated separately from two public datasets of cervical clinical LBC images.

**Figure 2:**
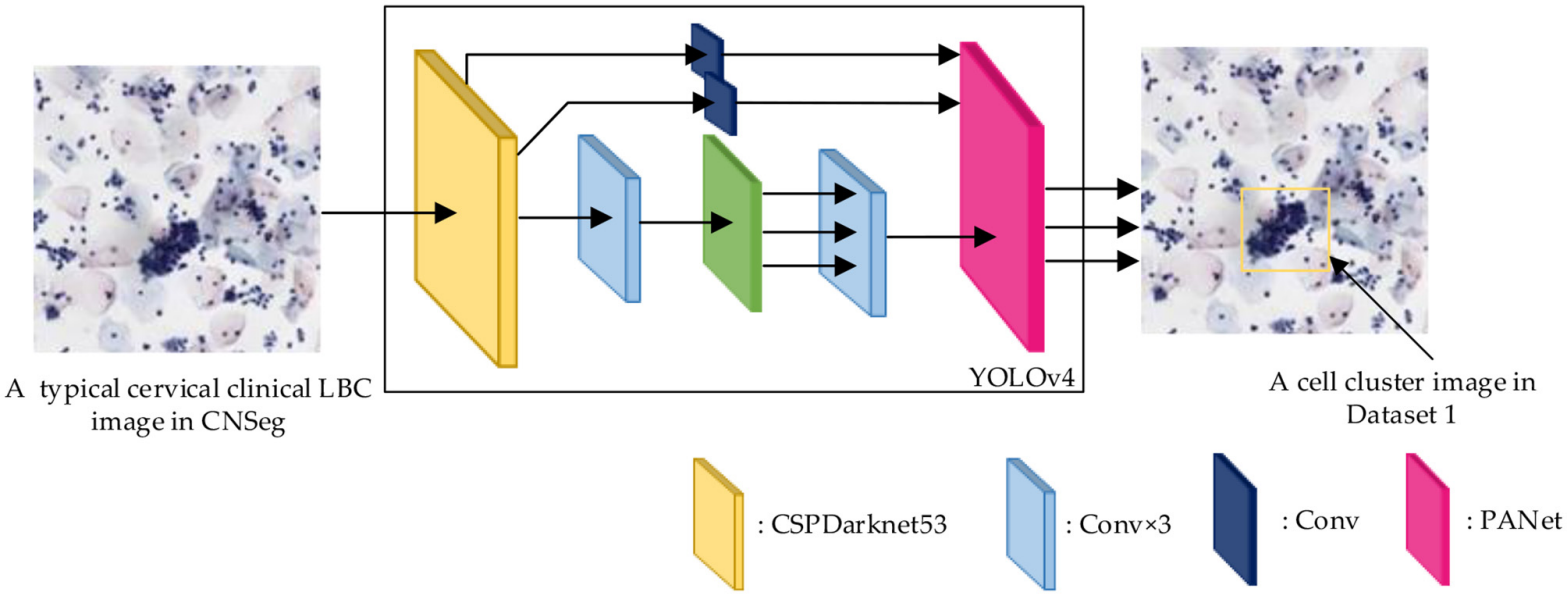
The generation process of a typical clinical cervical cell image in Dataset 1 based on YOLO-V4.

Table 1 reports the characteristics of Datasets 1 and 2. Specifically, the original dataset CNSeg includes 265 × 2048 × 2048 cervical clinical LBC images, provided by the 2nd Affiliated Hospital of Harbin Medical University, which is a set of clinical cervical cell images for LBC detection. First, Figure 2 shows that 2,363 × (150–500) × (150–500) cell cluster images (Dataset 1), marked by a yellow box, are generated by YOLO-V4 (Das et al., 2024). Then, 568 × (150–500) × (150–500) cell cluster images are randomly selected as the Test set (Test Set 1), and the remaining 1795 × (150–500) × (150–500) images are used as the training set. Furthermore, the training set of Dataset 1 includes 200 simple, 1500 normal, and 95 difficult cell cluster images. Meanwhile, to crop out more cervical clinical LBC images, a size of 224 × 224 is used to crop the 30 × 1,000 × 1,000 H&E-stained histological images obtained from different hospitals across multiple patients and organs of the original dataset MoNuSeg, resulting in 8,576 × 224 × 224 cropped H&E-stained histological images (Dataset 2). Then, 896 × 224 × 224 cropping of H&E-stained histological images is randomly selected as the Test set (Test set 2), and the remaining 7,680 × 224 × 224 images comprise the training set.

**Table 1:** Characteristics of Datasets 1 and 2.

**Dataset**	**Name**	**Number × cropped size**	**Train set**	**Test set**
Datasets 1	CNSeg (265 × 2,048 × 2,048)	2,363 × (150–500) × (150–500)	1,795 × (150–500) × (150–500)	568 × (150–500) × (150–500)
Datasets 2	MoNuSeg (30 × 1,000 × 1,000)	8,576 × 224 × 224	7,680 × 224 × 224	896 × 224 × 224

### 2.2 Methods

**Figure 3:**
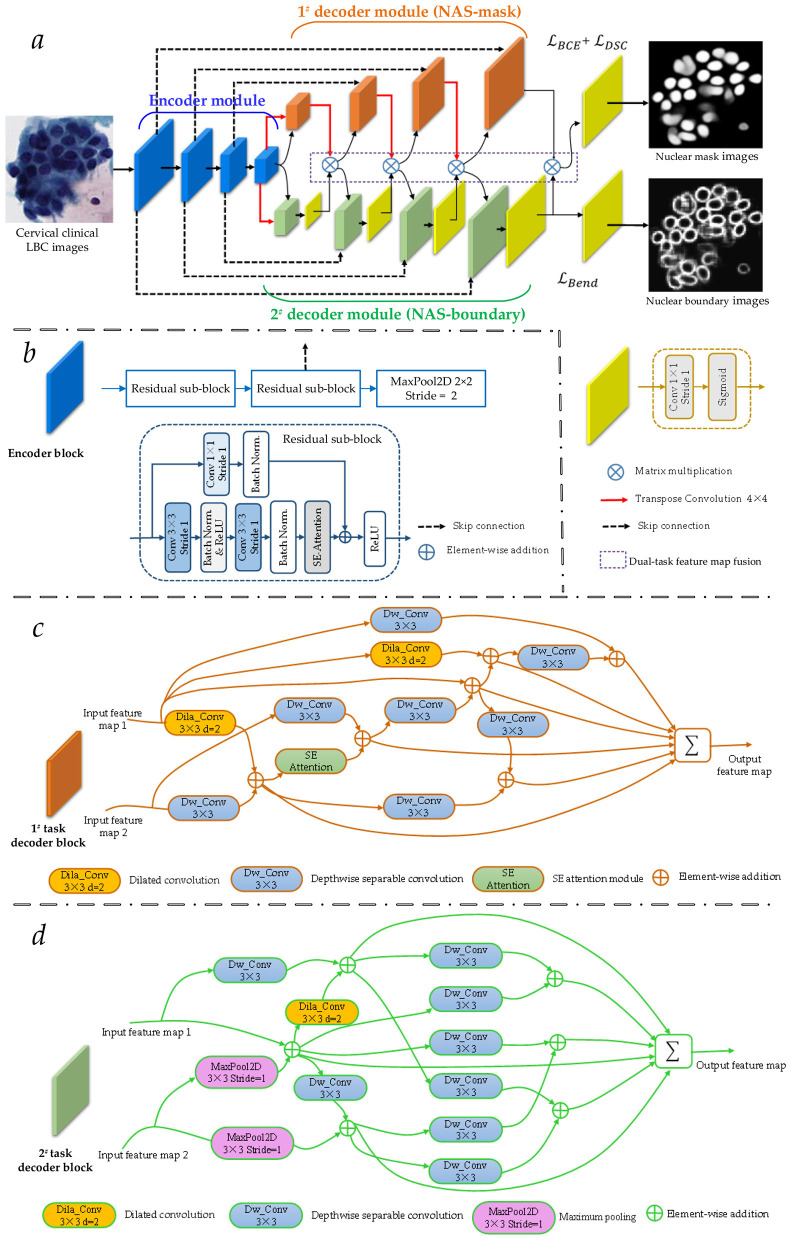
Detailed network architecture of the proposed Bloss-DDNet. **(a)** The overall network architecture of the proposed Bloss-DDNet; **(b)** Detailed network architecture of each encoder block in the shared encoder module; **(c)** Detailed network architecture of each 1^#^ task decoder block in the 1^#^ decoder module; **(d)** Detailed network architecture of each 2^#^ task decoder block in the 2^#^ decoder module.

Figure 3 shows the detailed network architecture of the proposed Bloss-DDNet. Specifically, due to Unet's outstanding performance in medical image segmentation (Yang et al., 2021; Zeng et al., 2023; Zaman et al., 2024; Sharma et al., 2025; Ronneberger et al., 2015; Yang et al., 2024a,b, 2025; Yingjian et al., 2025), the proposed Bloss-DDNet follows Unet's skip connection architecture to introduce low-level semantic information, helping to update gradients during backpropagation. Meanwhile, the proposed Bloss-DDNet considers the nuclear boundaries corresponding to high-level semantic information, avoiding the loss of boundary information as the network depth increases.

The overall network architecture of the proposed Bloss-DDNet is introduced as follows. Figure 3a further discloses the overall network architecture of the proposed Bloss-DDNet, which adds a boundary decoder branch for the cell nucleus boundary segmentation task. During the training stage of the proposed Bloss-DDNet, the added boundary decoder branch calculates the boundary loss to separate overlapping cell nuclei of cervical clinical LBC images. Specifically, the proposed Bloss-DDNet comprises a shared encoder module and two parallel dual-task decoder modules (1# and 2# decoder modules), which are connected to the output of the shared encoder module, thereby forming dual-task branches. The 1^#^ decoder module in the dual-task branches executes the cell nucleus mask segmentation task (1^#^ task). Meanwhile, the 2^#^ decoder module in the dual-task branches executes the cell nucleus boundary segmentation task (2^#^ task). Additionally, two feature maps, separately generated from each block of dual-task decoding branches, are fused through matrix multiplication (dual-task feature map fusion) to enhance the sensitivity to cell nucleus boundaries.

The network architecture of the shared encoder module, the 1^#^ and 2^#^ decoder modules, and their connections are introduced as follows. The shared encoder module consists of four sequentially connected encoder blocks, each with a blue color. Meanwhile, the 1^#^ and 2^#^ decoder modules in the dual-task branches also separately include four sequentially connected decoder blocks with orange and green colors. Specifically, the first three encoder blocks are separately connected to the last three decoder blocks of the 1^#^ and 2^#^ decoder modules via the skip connection. Meanwhile, the encoding feature map generated by the last encoder block of the shared encoder module separately connects the first decoder block of the 1# and 2# decoder modules after transposition convolution processing. Besides, to fuse the two decoding feature maps separately generated from each decoder block (1^#^ task decoder block with orange color and 2^#^ task decoder block with green color) of dual-task decoding branches, a 1 × 1 convolutional layer with a sigmoid function (a block with yellow color) is set behind each 2^#^ task decoder block. Before fusing the two decoding feature maps by matrix multiplication, the first three 1^#^ task decoder blocks are separately processed by a transposition convolution operation. Note that the decoding feature map generated by the last 1^#^ task decoder block is directly fused with the last decoding feature map generated by the last 2# task decoder block after a 1 × 1 convolutional layer with a sigmoid function, via matrix multiplication, to obtain the last 1^#^ task fused feature map. Lastly, the 1^#^ and 2^#^ last fused feature maps of the 1^#^ and 2^#^ decoder modules are separately processed by a 1 × 1 convolutional layer with a sigmoid function, generating the cell nucleus mask and the cell nucleus boundary segmentation images.

#### 2.2.1 Shared encoder module

As the encoder module's network depth increases, its performance will reach a specific limit even if the network gradient is effectively calculated (Hanin, 2018). When the encoder module's network reaches a certain depth, the gradient may become zero or reach a maximum value, causing it to disappear or explode (Hanin, 2018). Whether its gradient vanishes or explodes will affect the proposed Bloss-DDNet's performance. The problem of gradient vanishing or exploding can be solved by introducing skip connections in the residual blocks (Borawar and Kaur, 2023).

Figure 3b shows the detailed network architecture of each encoder block in the shared encoder module. Specifically, the shared encoder module comprises four sequentially connected encoder blocks, denoted by blue color. Each encoder block includes two sequentially connected residual sub-blocks and a 2D MaxPool layer with a 2 × 2 convolutional kernel (Stride = 2). Additionally, the main branch of each residual sub-block sequentially includes a 3 × 3 convolutional layer, a batch normalization (BN) layer with a ReLU activation function, a 3 × 3 convolutional layer, a BN layer, and an SE-Attention sub-block. Meanwhile, the side branch of each residual sub-block includes sequentially a 3 × 3 convolutional layer and a BN layer. Then, the attention feature map generated by the SE-Attention sub-block is fused with the BN feature map generated by the BN layer by element-wise addition. Introduced SE-Attention sub-block weights the BN feature maps of each channel in the main branch, creating more accurate encoding feature maps. This enhances the encoder module.

#### 2.2.2 1^#^ and 2^#^ decoder modules based on the NAS framework

Besides the encoder module, the decoder module is also crucial for the proposed Bloss-DDNet, as it directly affects the overlapping cell nucleus segmentation of cervical clinical LBC images. After fixing the encoder module, the neural architecture search (NAS) strategy is introduced to determine the network architecture of 1^#^ and 2^#^ decoder modules. Specifically, the NAS is initially used for classification tasks (Elsken et al., 2019). Howard et al. created a network for semantic segmentation in MobileNet V3 by modifying the classification network generated by NAS to shift the target to semantic segmentation operations for low-resolution images. Subsequently, Albert Shaw et al. proposed the SqueezeNAS method (Shaw et al., 2019), a search encoding framework for dense semantic segmentation network search, achieving good results. Inspired by its ideas, this article utilizes the NAS to search for 1# and 2# decoder modules, aiming to achieve high performance in cell nucleus segmentation.

**Figure 4:**
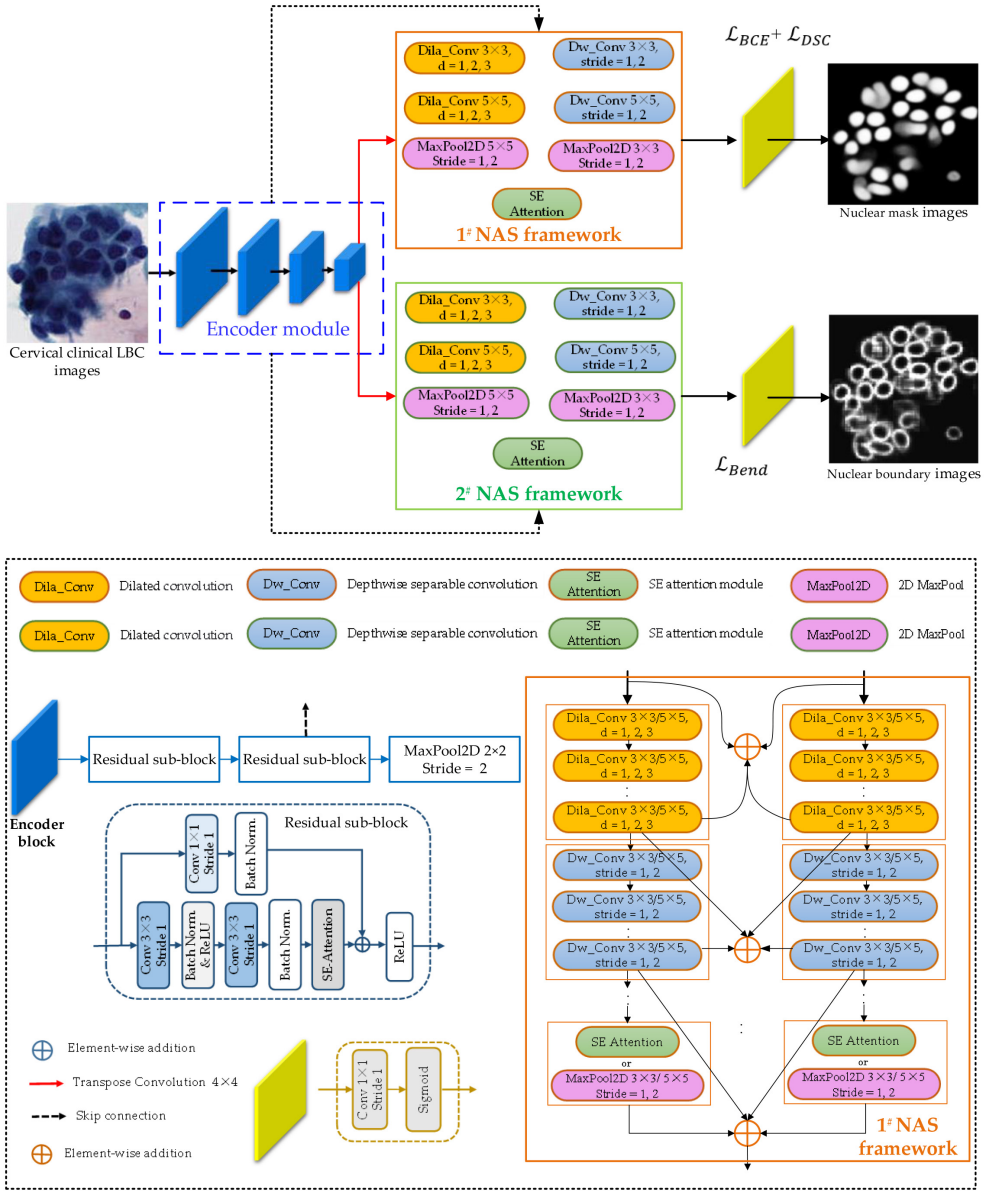
The detailed network architecture of the NAS for separately determining 1^#^ and 2^#^ decoder modules.

Figure 4 shows the detailed network architecture of NAS for separately determining 1^#^ and 2^#^ decoder modules (NAS-mask and NAS-boundary). Specifically, these decoder modules are separately determined based on the shared encoder module. Besides, the 1^#^ and 2^#^ NAS frameworks include the same sub-blocks. Therefore, only the specific search space of the 1^#^ NAS framework is given in Figure 4.

To fully utilize the advantages of dilated convolution, depthwise separable convolution, and SE Attention sub-blocks (Yu, 2015; Kaiser et al., 2017; Deng et al., 2020), the specific search space includes sequentially connected dilated convolution sub-blocks, depthwise separable convolution sub-blocks, SE Attention sub-blocks, and 2D MaxPool layers. The connections of these sub-blocks and layers are detailed in Figure 4. The convolutional kernel sizes of the dilated convolution, depthwise separable convolution, and 2D MaxPool layers are set to 3 × 3 and 5 × 5, respectively. The stride of the depthwise separable convolution and 2D MaxPool layer is set to 1 and 2, and the dilated convolution's dilation rate (d) is set to 1, 2, and 3. Lastly, following the above settings and connection relationships of the 1# NAS framework, various possible decoding networks are trained and tested on Dataset 1, and the optimal decoding network with the maximum Dice score is selected as the NAS-mask shown in Figure 3c. Similarly, following the above settings and connection relationships of the 2^#^ NAS framework, various possible decoding networks are also trained and tested on Dataset 1. The optimal decoding network is the one with the maximum Dice score, selected as the NAS-boundary, as shown in Figure 3d. Ultimately, the dilated convolution with a 3 × 3 kernel (*d* = 2 and Stride = 1), depthwise separable convolution with a 3 × 3 kernel (Stride = 1), and SE Attention sub-blocks are specified in the NAS-mask. In addition, the dilated convolution with 3 × 3 (*d* = 2 and Stride = 1), depthwise separable convolution with 3 × 3 (Stride = 1), and 2D MaxPool layer with 3 × 3 (Stride = 1) sub-blocks are determined in the NAS–boundary.

Specifically, 200 simple, 1,500 normal, and 95 difficult cell cluster images (224 × 224) in the training set of Dataset 1 are used to separately train various possible decoding networks of the 1# and 2# NAS frameworks based on the curriculum learning strategy (Bengio et al., 2009). Subsequently, 568 cell cluster images (448 × 448) in Test set 1 of Dataset 1 are used to separately test the trained decoding networks of the 1# and 2# NAS frameworks. Additionally, when various possible decoding networks are trained to obtain the NAS-mask, the loss functions are configured as the NAS-mask loss function L_NAS-mask_, which includes the binary cross-entropy loss L_*BCE*_ and Dice loss L_*DSC*_ (Zeng et al., 2023; Zaman et al., 2024; Yang et al., 2024a,b). The weight coefficients of the binary cross-entropy loss L_*BCE*_ and Dice loss L_*DSC*_ are set to 0.3 and 0.7, respectively. The binary cross-entropy loss L_*BCE*_ and the Dice loss L_*DSC*_ are shown in Equations 1, 2. When various possible decoding networks are trained to obtain the NAS-boundary, the loss functions are configured as the NAS-boundary loss function (bending loss, as shown in Equation 5).


$$\begin{array}{r} {\text{L}}_{BCE}=f_{Loss-BCE}\left(y_{i},p_{i}\right)\\ =-\left[y_{i}•\log\left(p_{i}\right)+\left(1-y_{i}\right)•\log\left(1-p_{i}\right)\right]\\ {\text{L}}_{\text{DCS}}={\text{f}}_{\text{Loss-DCs}}\left(y_{i},p_{i}\right) \end{array}$$ (1)

$$\begin{array}{c} =1-\left[2\underset{i}{\sum }\left[y_{i}p_{i}\right]/\left(\underset{i}{\sum }\left[y_{i}^{2}\right]+\underset{i}{\sum }\left[p_{i}^{2}\right]\right)\right], \end{array}$$ (2)

here, *y*_*i*_ and *p*_*i*_ represents pixel value in the *i*-th ground truth image *Y* and the pixel value in the *i*-th cervical clinical LBC image *P* being classified as the cell nucleus, respectively.

#### 2.2.3 Bending loss

This study's loss function design introduced the concept of curvature to describe overlapping nucleus boundaries. Curvature is a physical quantity used to indicate the degree of curvature of a curve at a certain point (Federer, 1959). Meanwhile, the curvature of a curve is directly proportional to its bending degree. Specifically, the larger the curvature of a curve, the greater its bending degree, and vice versa.

**Figure 5:**
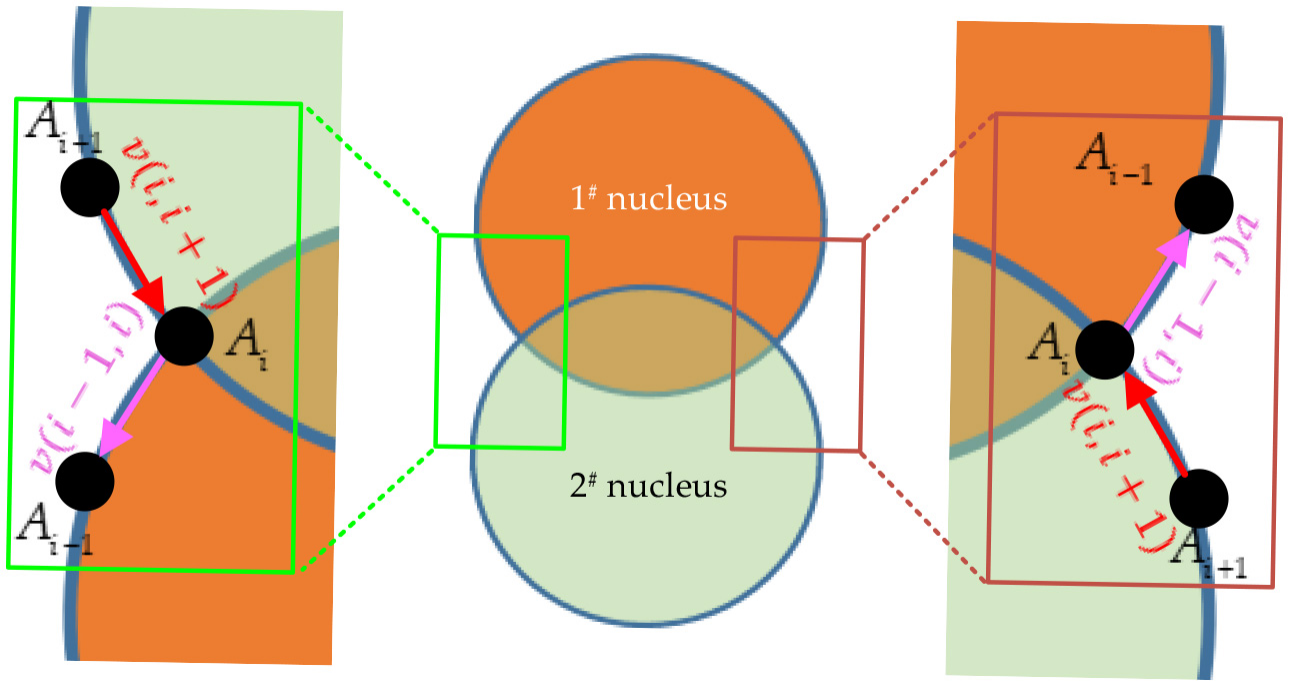
Edge vector representation at the intersections of overlapping nuclei.

Figure 5 shows the edge vector representation of the intersections of the overlapping nucleus. In cervical clinical LBC images, the curvature of a nucleus boundary changes smoothly. However, if two or more nuclei (such as a 1# nucleus and a 2^#^ nucleus) have overlapping boundaries, the intersections *A*_*i*_(*x*_*i*_, *y*_*i*_) on the boundaries of these nuclei will exhibit noticeable changes in curvature. Inspired by this observation, the curvature changes of the nucleus boundaries can comprehensively reflect the boundary position of the overlapping nuclei. Furthermore, the detailed construction process of this bending loss is as follows.

First, the adjacent pixel points *A*_*i*−1_(*x*_*i*−1_, *y*_*i*−1_) and *A*_*i*+1_(*x*_*i*+1_, *y*_*i*+1_) at both sides of this intersection *A*_*i*_(*x*_*i*_, *y*_*i*_) on each nucleus boundary are determined. Second, the first side vector, denoted by a purple arrow*v*(*i*−1, *i*) = *A*_*i*_(*x*_*i*_, *y*_*i*_)−*A*_*i*−1_(*x*_*i*−1_, *y*_*i*−1_), is constructed from the pixel point *A*_*i*−1_(*x*_*i*−1_, *y*_*i*−1_) to the intersection *A*_*i*_(*x*_*i*_, *y*_*i*_). Meanwhile, the second side vector, denoted by a red arrow *v*(*i, i*+1) = *A*_*i*+1_(*x*_*i*+1_, *y*_*i*+1_)−*A*_*i*_(*x*_*i*_, *y*_*i*_), is constructed from the intersection *A*_*i*_(*x*_*i*_, *y*_*i*_) to the pixel point*A*_*i*+1_(*x*_*i*+1_, *y*_*i*+1_). Third, the curvature *k*_*i*_ at the intersection *A*_*i*_(*x*_*i*_, *y*_*i*_) can be calculated based on the first side vector *v*(*i*−1, *i*) and the second side vector *v*(*i, i*+1). Then, the discrete bending loss *BE*(*i*) of the intersection *A*_*i*_(*x*_*i*_, *y*_*i*_) is determined based on the curvature *k*_*i*_. Lastly, the bending loss L_*Bend*_ is introduced based on the discrete bending loss *BE*(*i*).

Based on the above, the specific implementation details are represented by mathematical Equations 3–5:


$$\begin{array}{c} k\left(i\right)=\frac{2|v\left(i-1,i\right)\times v\left(i,i+1\right)|}{|v\left(i-1,i\right)||v\left(i-1,i\right)|+v\left(i-1,i\right)\cdot v\left(i,i+1\right)} \end{array}$$ (3)

$$\begin{array}{c} \text{BE}\left(i\right)=\frac{k{\left(i\right)}^{2}}{\left|v\left(i,i+1\right)|+|v\left(i-1,i\right)\right|} \end{array}$$ (4)

$$\begin{array}{c} {\text{L}}_{\text{Bord}}={\text{f}}_{\text{Loss-Bend}}\left(\text{BE}\left(i\right)\right)=\frac{1}{m}\overset{m}{\underset{i=1}{\sum }}\text{BE}\left(i\right), \end{array}$$ (5)

where symbol |^*^| represents the length of a vector; symbol × represents the vector multiplication operation; symbol · represents the vector dot multiplication operation;*v*(*i*−1, *i*) represents the first side vector from the pixel point *A*_*i*−1_(*x*_*i*−1_, *y*_*i*−1_) to the intersection *A*_*i*_(*x*_*i*_, *y*_*i*_); *v*(*i, i*+1) represents the second side vector from the intersection *A*_*i*_(*x*_*i*_, *y*_*i*_) to the pixel point*A*_*i*+1_(*x*_*i*+1_, *y*_*i*+1_); *k*_*i*_ represents the curvature at the intersection *A*_*i*_(*x*_*i*_, *y*_*i*_);*BE*(*i*) represents the discrete bending loss of the intersection *A*_*i*_(*x*_*i*_, *y*_*i*_); L_*Bend*_ represents the bending loss of the discrete bending loss*BE*(*i*); *m* represents the number of pixel points on the nucleus boundary.

## 3 Experiments and results

This section comprehensively implements the ablation study and comparative experiment. Then, the overlapping cell nucleus segmentation results based on the cervical clinical LBC images are presented.

### 3.1 Experiments

This section includes the ablation study, comparative experiment, and their evaluation metrics. Additionally, the experimental configuration of all experiments and the operation of the NAS framework for determining the 1^#^ and 2^#^ decoder modules in this study are consistent. First, all network architectures uniformly adopt the PyTorch 1.8 framework. Second, the resolution of Train sets 1 and 2 is resized to 448 × 448 before training these networks. Third, the NVIDIA V100 Tensor Core 40G GPU and Intel(R) Xeon(R) Gold 5218 2.30 GHz CPU were used during the training process for these networks. Additionally, during network training, the batch size is set to 8, and the Adam optimizer is used with a preset learning rate of 1e^−4^. Lastly, Test sets 1 and 2 of Dataset 1 and Dataset 2, respectively, include 568 × (150–500) × (150–500) cell cluster images and 896 × 224 × 224 cropping of H&E-stained histological images. Additionally, the size of the Test sets should be uniformly resized to 448 × 448 to ensure consistency with the Training sets.

#### 3.1.1 Ablation study

**Figure 6:**
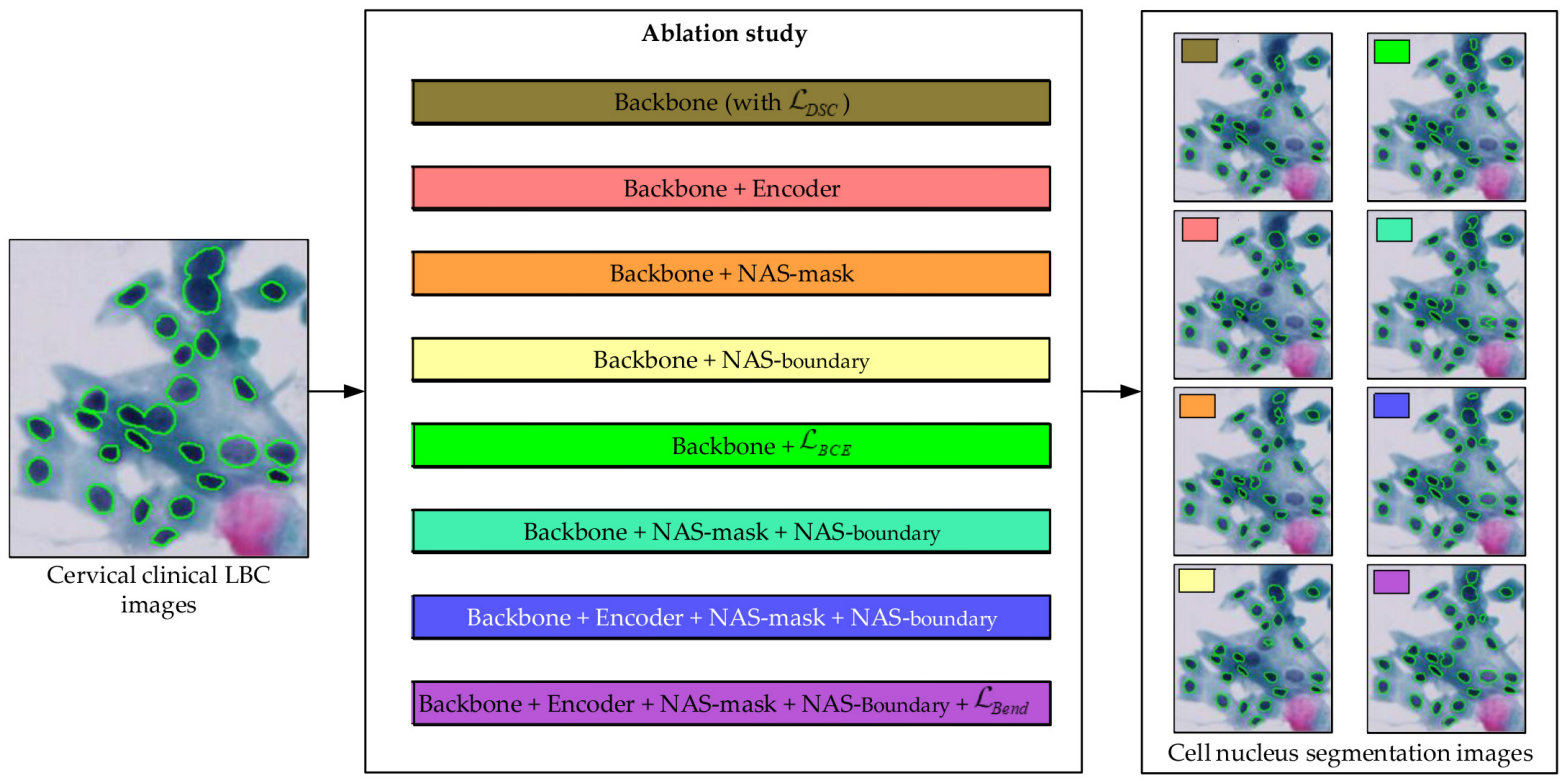
Ablation study design of the proposed BLoss-DDNet (Backbone + Encoder + NAS-mask + NAS-boundary +L_*Bend*_).

To reasonably verify the performance of the network structure of the proposed BLoss-DDNet (Backbone + Encoder + NAS-mask + NAS-boundary +L_*Bend*_), an ablation study on Dataset 1 is conducted in this paper. Figure 6, Table 2 report the specific design of the ablation study.

**Table 2:** The specific design of the ablation study.

**Network**	**Shared encoder**	**NAS-mask**	**NAS-boundary**	**L_*BCE*_**	**L_*DSC*_**	**L_*Bend*_**
Backbone					√	
Backbone + Encoder	√				√	
Backbone + NAS-mask		√			√	
Backbone + NAS-boundary			√		√	
Backbone +L_*BCE*_				√	√	
Backbone + NAS-mask + NAS-boundary		√	√	√	√	
Backbone + Encoder + NAS-mask + NAS-boundary	√	√	√	√	√	
Backbone + Encoder + NAS-mask + NAS-boundary +L_*Bend*_	√	√	√	√	√	√

Specifically, based on the basic architecture of U-Net, the encoder module and two decoder modules (the 1^#^ and 2^#^ decoder modules) in Figure 3a of this Backbone network adopt Unet's original modules. Meanwhile, the original loss function L_*DSC*_is used during training of the Backbone network.

Compared to the Backbone architecture, the Backbone + Encoder replaces Unet's original encoder module with the shared encoder module to encode the cervical clinical LBC images. Besides, the Backbone + NAS-mask and Backbone + NAS-boundary separately replace Unet's original decoder module with the NAS-mask and NAS-boundary to decode the decoding feature map generated by Unet's original encoder module. Meanwhile, the Backbone + NAS-mask + NAS-boundary replaces Unet's original decoder module with the NAS-mask and NAS-boundary. The Backbone + Encoder + NAS-mask + NAS-boundary replaces Unet's original encoder and decoder module with the shared encoder module, NAS-mask, and NAS-boundary.

Compared to the loss function, the dual-task branches of the Backbone, Backbone + Encoder, Backbone + NAS-mask, and Backbone + NAS-boundary all utilize the same loss function L_*DSC*_. Additionally, the dual-task branches of Backbone +L_*BCE*_, Backbone + NAS-mask + NAS-boundary, and Backbone + Encoder + NAS-mask + NAS-boundary utilize the same loss function L_*DSC*_+ L_*BCE*_. The weight coefficients of the binary cross-entropy loss L_*BCE*_ and Dice lossL_*DSC*_ are set to 0.3 and 0.7, respectively. Lastly, the dual-task branches of the Backbone + Encoder + NAS-mask + NAS-boundary +L_*Bend*_ separately configure the loss function L_*DSC*_+ L_*BCE*_ and L_*Bend*_. Specifically, the loss function of the Backbone + Encoder + NAS-mask + NAS-boundary +L_*Bend*_ is shown in Equation 6.


$$\begin{array}{c} \text{ L}={\text{L}}_{\text{NAS-mask}}+{\text{L}}_{\text{NAS-boundary}}=\left(\alpha {\text{L}}_{\text{BCE}}+\beta {\text{L}}_{\text{DSC}}\right)+\gamma {\text{L}}_{\text{Bend}}, \end{array}$$ (6)

where α = 0.4, β = 0.3, and γ = 0.3.

#### 3.1.2 Comparative experiment

**Figure 7:**
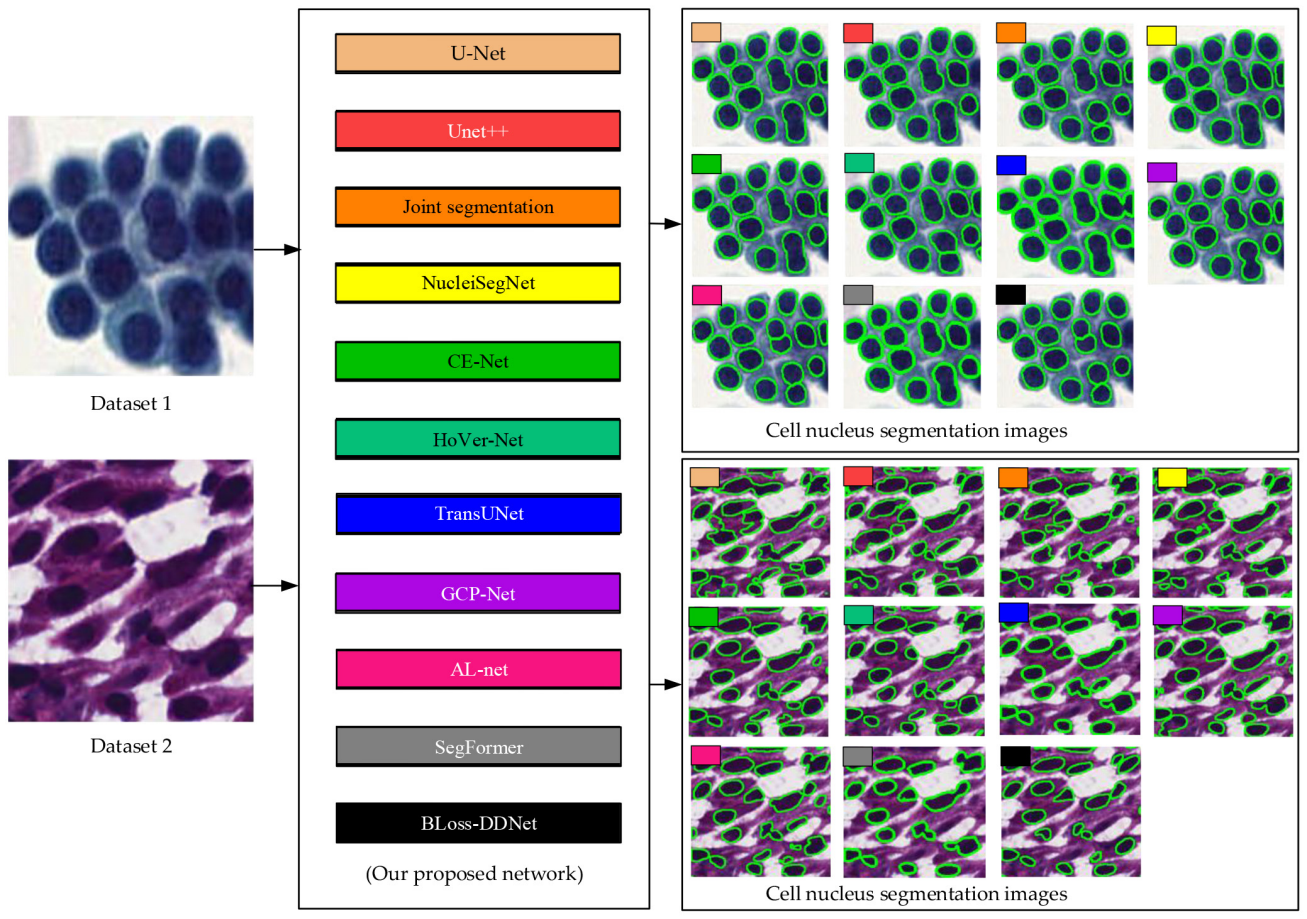
Experiment design for comparing the proposed BLoss-DDNet and other existing medical image segmentation networks.

Figure 7 shows the specific design of the comparative experiment. Specifically, this section separately evaluates the proposed Bloss-DDNet on Dataset 1 and Dataset 2, comparing the classic U-Net (Ronneberger et al., 2015) and Unet++ (Zhou et al., 2018) models for medical image segmentation, Joint segmentation (Qu et al., 2019) model for kernel segmentation, NucleiSegNet (Lal et al., 2021), CE Net (Gu et al., 2019) network, HoVer Net (Graham et al., 2019) network for tissue cytology segmentation, TransUNet (Chen et al., 2102), GCP-Net (Yang et al., 2022), AL Net (Zhao et al., 2021), and SegFormer (Eskandari et al., 2023). Meanwhile, the same framework, training set, and training strategy are used for all networks, along with the same Test set, to ensure fairness in the comparison.

#### 3.1.3 Evaluation metrics

To assess the performance differences in the ablation study and the comparative experiment, three standard evaluation metrics are used in this study: the Aggregated Jaccard Index (AJI) (Kumar et al., 2017), the Dice coefficient (Dice) (Dice, 1945), and the Panoptic Quality (PQ) (Kirillov et al., 2019). The specific evaluation metrics are represented by mathematical Equations 7, 9:


$$\begin{array}{l} \text{AJI}=\overset{n}{\underset{i=1}{\sum }}G_{i}\cap P_{j}\text{/}(\overset{n}{\underset{i=1}{\sum }}G_{i}\cup P_{j}+\underset{k\in U}{\sum }P_{k}), \end{array}$$ (7)

$$\begin{array}{c} \text{Dice}=2\times |G\cap P|\text{/}\left(\left|G|+|P\right|\right), \end{array}$$ (8)

$$\begin{array}{c} \text{PQ}=\sum_{(p,g)\in \text{TP}}\text{IoU}(p,g)/\left(\left|\text{TP}|+\frac{1}{2}|\text{FP}|+\frac{1}{2}|\text{FN}\right|\right), \end{array}$$ (9)

where G_i_ is the i-th nucleus in a ground truth with a total of N nuclei. P_k_ means the k-th connected component in prediction, which has the largest Jaccard Index with G_i_, and each k can only be used once. U is the connected component that does not correspond to the ground truth in the prediction. FP represents the over-segmented region, TP represents the accurately segmented region, FN represents the under-segmented region, and TN represents the region accurately segmented into the background. Each (p,g) indicates a pair of mask-accurate positive detections and their corresponding ground truth. Note that a mask prediction can only be regarded as the true positive when IOU(p,g) > 0.5.

### 3.2 Results

This section presents the results of the ablation study and comparative experiment, highlighting the performance of the proposed BLoss-DDNet.

#### 3.2.1 Ablation results

**Figure 8:**
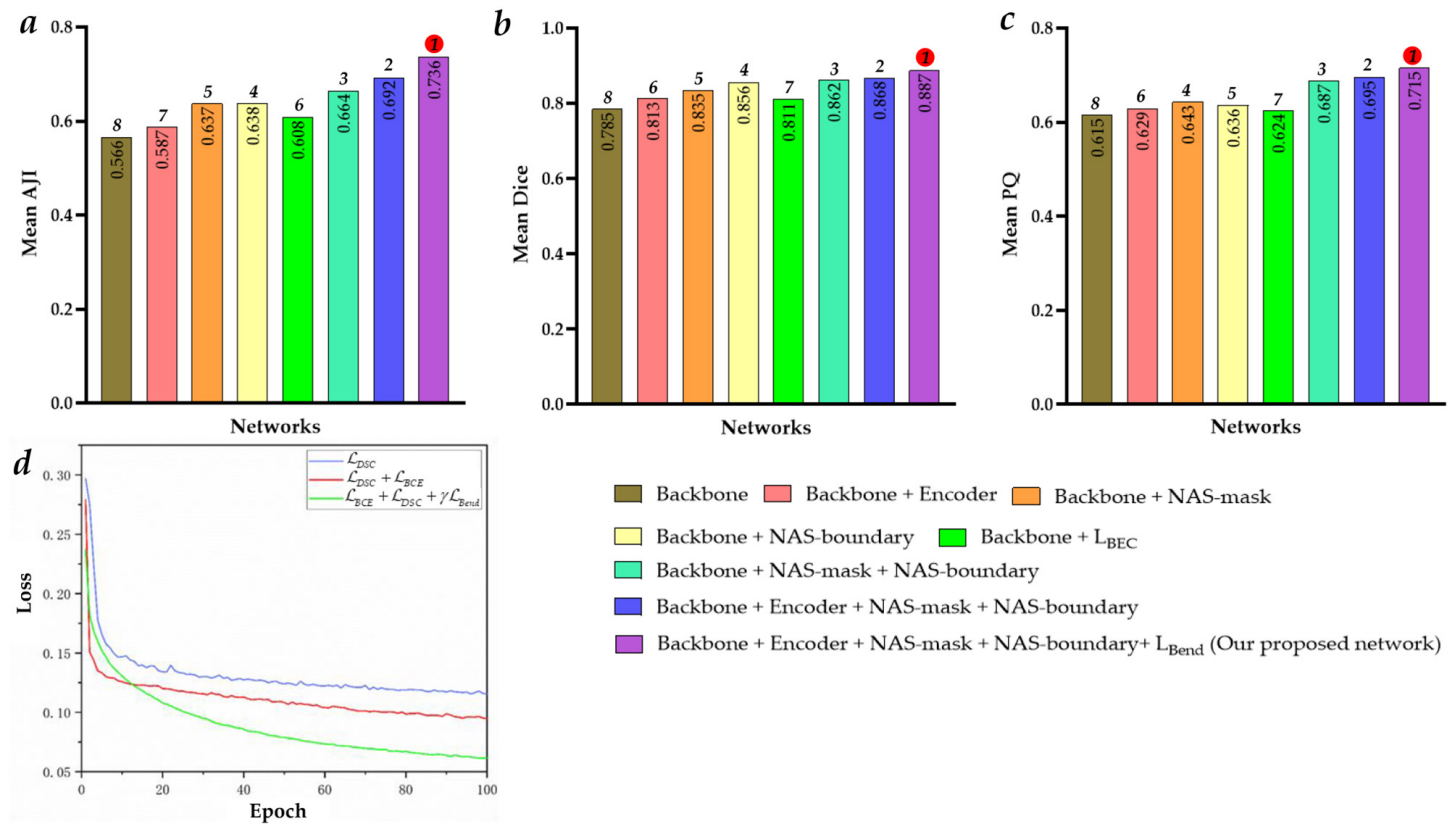
Ablation study results of the proposed BLoss-DDNet on Test set 1. **(a)** Mean AJI; **(b)** Mean Dice; **(c)** Mean PQ; **(d)** Loss curves.

**Figure 9:**
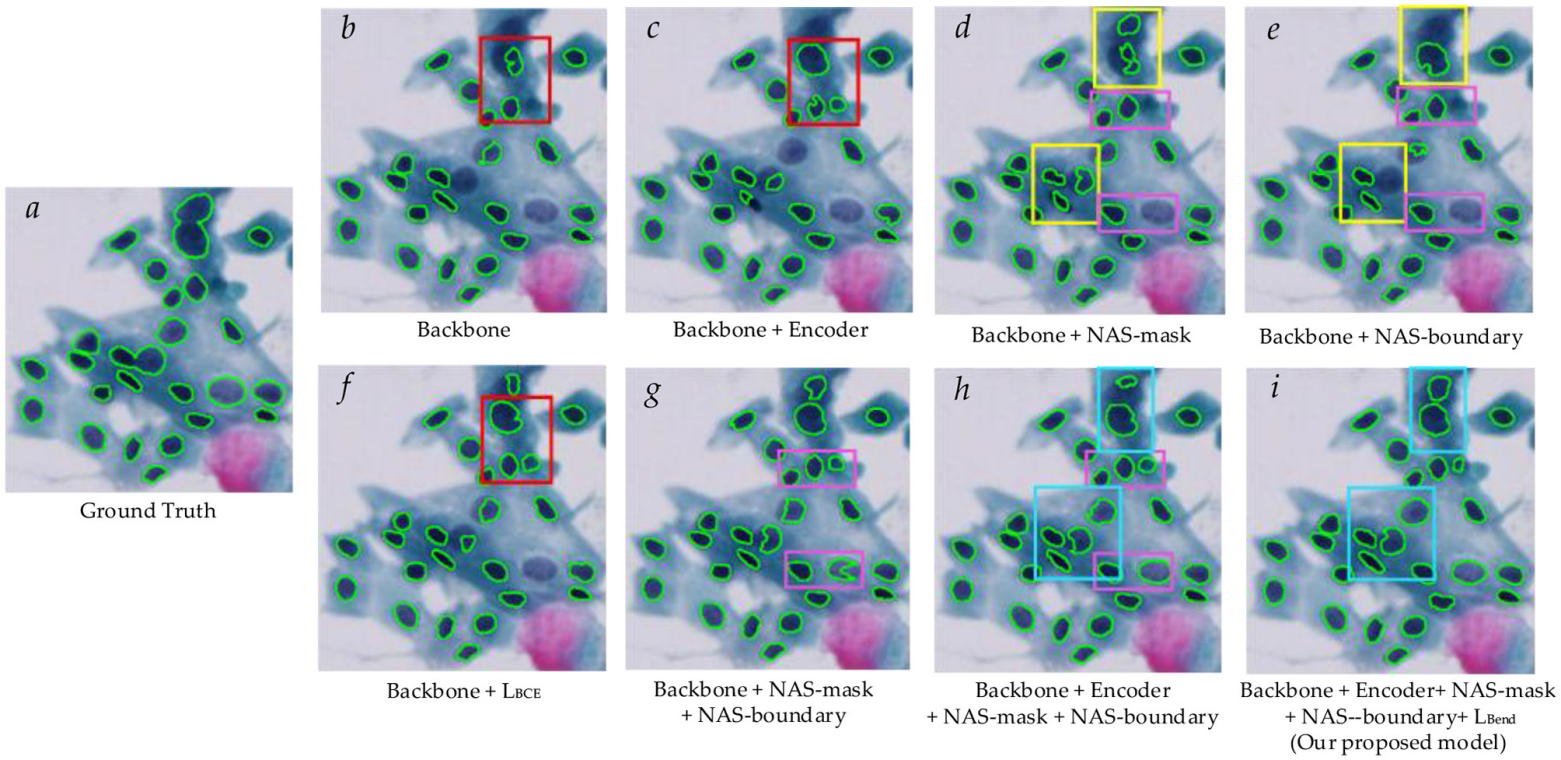
Typical visualized ablation study results of the proposed BLoss-DDNet on Test set 1. **(a)** Ground Truth; **(b)** Backbone; **(c)** Backbone +Encoder; **(d)** Backbone + NAS-mask; **(e)** Backbone + NAS-boundary; **(f)** Backbone +L_*BCE*_; **(g)** Backbone + NAS-mask + NAS-boundary; **(h)** Backbone + Encoder + NAS-mask + NAS-boundary; **(i)** Backbone + Encoder + NAS-mask + NAS-boundary +L_*Bend*_.

Figure 8 shows the ablation study results of the proposed BLoss-DDNet (Backbone + Encoder + NAS-mask + NAS-boundary +L_*Bend*_) on Test set 1 of Dataset 1. Overall, our proposed BLoss-DDNet performs the best in all evaluation metrics, achieving a mean AJI of 73.6%, a mean Dice of 88.7%, and a mean PQ of 71.5%. Meanwhile, Figure 9 shows the typical visualized ablation study results of the proposed BLoss-DDNet on Test set 1.

First, the effectiveness of the shared encoder module is compared. Compared to the Backbone, the mean AJI, the mean Dice, and the mean PQ of the Backbone + Encoder have improved by 2.1%, 2.8%, and 1.4%, respectively. In addition, compared to Figures 9b, c shows that cell nucleus segmentation with the red box has been improved. Similarly, compared to the Backbone + NAS-mask + NAS-boundary, the mean AJI, the mean Dice, and the mean PQ of the Backbone + Encoder + NAS-mask + NAS-boundary have improved by 2.8%, 0.6%, and 0.8%, respectively.

Second, the effectiveness of the NAS mask and the NAS boundary is compared. Compared to the Backbone, the mean AJI, the mean Dice, and the mean PQ of the Backbone + NAS-mask have improved by 7.1%, 5.0%, and 2.8%, respectively. Besides, compared to the Backbone, the mean AJI, the mean Dice, and the mean PQ of the Backbone + NAS-boundary have improved by 7.2%, 7.1%, and 2.1%, respectively. Meanwhile, compared to the Backbone + NAS-mask, the mean AJI, the mean Dice, and the mean PQ of the Backbone + NAS-mask + NAS-boundary have improved by 2.7%, 2.7%, and 4.4%, respectively. Besides, compared to the Backbone + NAS-boundary, the mean AJI, the mean Dice, and the mean PQ of the Backbone + NAS-mask + NAS-boundary have improved by 2.6%, 0.6%, and 5.1%, respectively. Especially compared to the Backbone, the mean AJI, the mean Dice, and the mean PQ of the Backbone + NAS-mask + NAS-boundary have improved by 9.8%, 7.7%, and 7.2%, respectively. In addition, compared to Figures 9b, d, e, g show that cell nucleus segmentation with the pink boxes has been improved. Compared to Figures 9g, h shows that cell nucleus segmentation with the pink boxes has been further improved. Besides, the segmenting performance of the cell nucleus with the yellow boxes in Figure 9d is better than that in Figure 9e.

Lastly, the effectiveness of the loss function is compared. Compared to the Backbone (with L_*DSC*_), the mean AJI, the mean Dice, and the mean PQ of the Backbone (with L_*DSC*_) + L_*BCE*_ have improved by 4.2%, 2.6%, and 0.9%, respectively. Besides, compared to the Backbone + Encoder + NAS-mask + NAS-boundary (with L_*DSC*_ + L_*BCE*_), the mean AJI, the mean Dice, and the mean PQ of the Backbone + Encoder + NAS-mask + NAS-boundary (with L_*DSC*_ + L_*BCE*_) + L_*Bend*_ have improved by 4.4%, 1.9%, and 2.0%, respectively. In addition, Figure 9e shows the loss curves of the training process of the Backbone (with L_*DSC*_), the Backbone (with L_*DSC*_) + L_*BCE*_, and the Backbone + Encoder + NAS-mask + NAS-boundary (with L_*DSC*_ + L_*BCE*_) + L_*Bend*_. The Backbone (with L_*DSC*_)'s loss curve, marked in blue, initially decreases significantly and then gradually decreases after about the 8th epoch. Compared to the Backbone (with L_*DSC*_)'s loss curve, the Backbone (with L_*DSC*_) + L_*BCE*_‘s loss curve, marked in red, more quickly decreases at the beginning of the training, gradually decreases after about the 5th epoch, and converges to a smaller loss. Compared to the Backbone (with L_*DSC*_) + L_*BCE*_‘s loss curve, the Backbone + Encoder + NAS-mask + NAS-boundary (with L_*DSC*_ + L_*BCE*_) + L_*Bend*_‘s loss curve, marked in green, initially decreases. Then, it gradually decreases after about the 12th epoch and converges to a smaller loss. Meanwhile, the segmenting performance of the cell nucleus with the red box in Figure 9f is better than that in Figure 9b. Further, the segmenting performance of the cell nucleus with the blue boxes in Figure 9i is also better than that in Figure 9h.

#### 3.2.2 Comparative results

**Figure 10:**
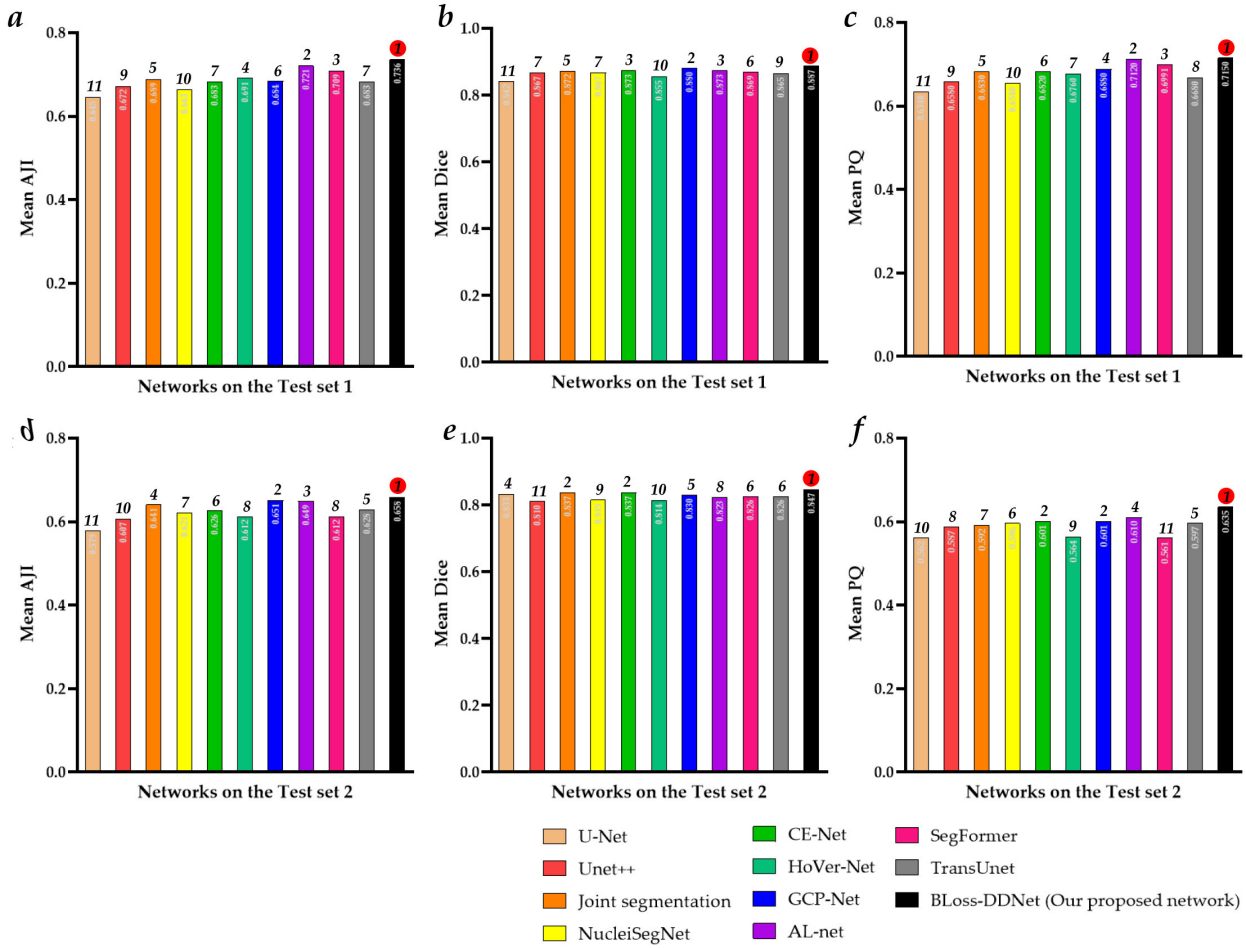
Comparative results on Test sets 1 and 2. **(a)** Mean AJI of Test set 1; **(b)** Mean Dice of Test set 1; **(c)** Mean PQ of Test set 1; **(d)** Mean AJI of Test set 2; **(e)** Mean Dice of Test set 2; **(f)** Mean PQ of Test set 2.

Table 3, Figure 10 present the comparative experimental results of the proposed BLoss-DDNet and other existing medical image segmentation networks on Test sets 1 and 2 of their respective Datasets 1 and 2.

**Table 3:** The comparative experiment results of Test sets 1 and 2.

**Network**	**Test set 1**			**Test set 2**		
	**Mean AJI** ↑	**Mean Dice** ↑	**Mean PQ** ↑	**Mean AJI** ↑	**Mean Dice** ↑	**Mean PQ** ↑
U-Net [29]	0.645	0.842	0.634	0.579	0.833	0.562
Unet++ [18]	0.672	0.867	0.658	0.607	0.810	0.587
Joint segmentation [20]	0.689	0.872	0.683	0.641	0.837	0.592
NucleiSegNet [21]	0.663	0.867	0.654	0.621	0.815	0.596
CE-Net [19]	0.683	0.873	0.682	0.626	0.837	0.601
HoVer-Net [43]	0.691	0.855	0.676	0.612	0.814	0.564
TransUNet [24]	0.683	0.865	0.668	0.628	0.826	0.597
GCP-Net [4]	0.684	0.880	0.688	0.651	0.830	0.601
AL-net [22]	0.721	0.873	0.712	0.649	0.823	0.610
SegFormer [25]	0.709	0.869	0.699	0.612	0.826	0.561
BLoss-DDNet (proposed)	0.736	0.887	0.715	0.658	0.847	0.635

**Figure 11:**
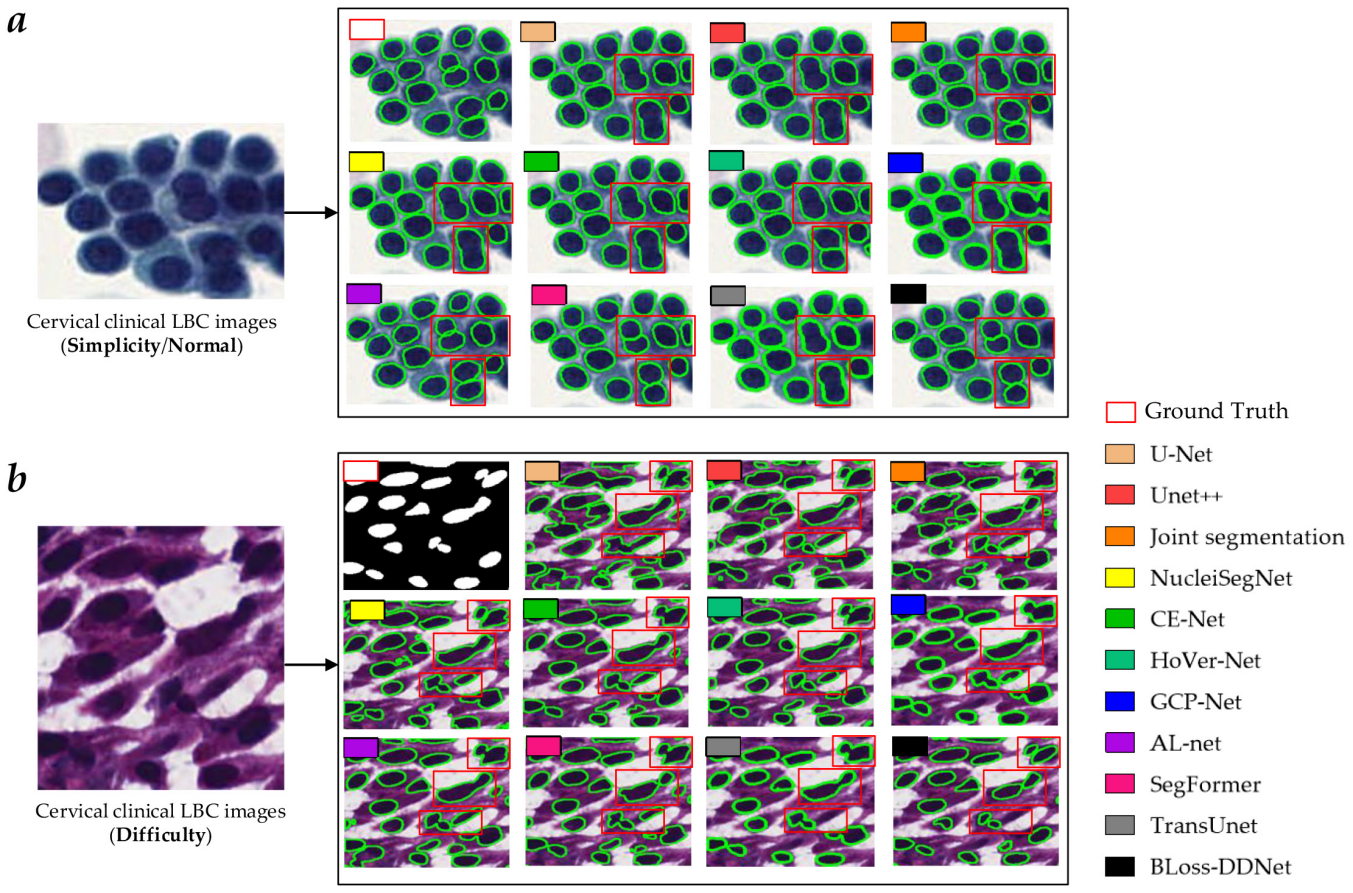
Typical visualized comparative results on Test sets 1 and 2. **(a)** Typical visualized comparative results on Test set 1; **(b)** Typical visualized comparative results on Test set 2.

Overall, our proposed BLoss-DDNet performs the best in all evaluation metrics on Test set 1, achieving a mean AJI of 73.6%, a mean Dice of 88.7%, and a mean PQ of 71.5%. Additionally, our proposed BLoss-DDNet performs the best in all evaluation metrics on Test set 2, achieving a mean AJI of 65.8%, a mean Dice of 84.7%, and a mean PQ of 63.5%. Meanwhile, Figure 11 shows the typical visualized ablation study results of the proposed BLoss-DDNet on Test sets 1 and 2.

Compared to the U-Net, Unet++, Joint segmentation, NucleiSegNet, CE Net, HoVer Net, TransUNet, GCP-Net, AL Net, and SegFormer, the mean AJI of the proposed BLoss-DDNet on Test set 1 has improved by 9.1%, 6.4%, 4.7%, 7.3%, 5.3%, 4.5%, 5.3%, 5.2%, 1.5%, and 2.7%, respectively. Additionally, compared to the networks mentioned above, the mean Dice of the proposed BLoss-DDNet on Test set 1 has shown improvements of 4.5%, 2.0%, 1.5%, 2.0%, 1.4%, 3.2%, 2.2%, 0.7%, 1.4%, and 1.8%, respectively. Lastly, compared to these networks above, the mean PQ of the proposed BLoss-DDNet on Test set 1 has improved by 8.1%, 5.7%, 3.2%, 6.1%, 3.3%, 3.9%, 4.7%, 2.7%, 0.3%, and 1.6%, respectively. Meanwhile, compared to the U-Net, Unet++, Joint segmentation, NucleiSegNet, CE Net, HoVer Net, TransUNet, GCP-Net, AL Net, and SegFormer, the mean AJI of the proposed BLoss-DDNet on Test set 2 has also improved by 7.9%, 5.1%, 1.7%, 3.7%, 3.2%, 4.6%, 3.0%, 0.7%, 0.9%, and 4.6%, respectively. Besides, compared to the networks above, the mean Dice of the proposed BLoss-DDNet on Test set 2 has also improved by 1.4%, 3.7%, 1.0%, 3.2%, 1.0%, 3.3%, 2.1%, 1.7%, 2.4%, and 2.1%, respectively. Finally, compared to these networks above, the mean PQ of the proposed BLoss-DDNet on Test set 2 has also improved by 7.3%, 4.8%, 4.3%, 3.9%, 3.4%, 7.1%, 3.8%, 3.4%, 2.5%, and 7.4%, respectively.

#### 3.2.3 Computation time and space requirements

Table 4 reports the computation time and space requirements of the proposed BLoss-DDNet and other existing medical image segmentation networks. Specifically, four standard indicators were used to evaluate the computational time and space requirements of the proposed BLoss-DDNet and other existing medical image segmentation networks, including Floating-Point Operations Per Second (FLOPS), parameters, training time, and test time. In addition, when calculating all standard indicators of computation time and space requirements, all networks use the same hardware configuration and hyperparameters. The computation time and space requirements of the proposed BLoss-DDNet are as follows: FLOPS of 119.88 G, parameters of 46.01 M, training time of 2.4 seconds per iteration (s/iteration), and test time of 11.3 seconds per image (s/image).

**Table 4:** The computation time and space requirements.

**Network**	**FLOPS (G)**	**Parameters (M)**	**Training time (s/iteration)**	**Test time (s/image)**
U-Net [29]	449.91	37.66	2.5	15.8
Unet++ [18]	225.22	44.01	2.8	11.1
Joint segmentation [20]	49.28	24.40	1.9	13.9
NucleiSegNet [21]	86.89	5.89	2.1	21.8
CE-Net [19]	53.24	29.00	2.3	14.4
HoVer-Net [43]	96.62	48.72	2.5	12.7
TransUNet [24]	66.24	10.63	2.6	9.1
GCP-Net [4]	117.16	52.37	1.7	8.9
AL-net [22]	102.22	27.44	2.8	11.6
SegFormer [25]	17.16	22.27	2.6	13.5
BLoss-DDNet (proposed)	119.88	46.01	2.4	11.3

## 4 Discussion

This section conducts the following discussions based on the experimental results. Additionally, this section highlights the study's limitations and outlines its future direction.

### 4.1 Dual-task decoding branches

One of the reasons why our proposed Bloss-DDNet is effective is attributed to the dual-task decoding branches. The dual-task decoding branches, which incorporate fast remote dependency relationships and global context connections, are constructed using the NAS-mask and NAS-boundary decoders in the backbone network, along with a shared encoder. Specifically, based on the skip connections in residual and SE-Attention sub-blocks to alleviate the problems of gradient vanishing or exploding (Hanin, 2018; Borawar and Kaur, 2023), the NAS-mask and NAS-boundary focus on the cell nucleus segmentation of masks and boundaries, respectively, forming a dual-task strategy. Therefore, this dual task strategy adopts two effective dimensions, mask and boundary dimensions, to segment the masks and boundaries of the cell nucleus separately.

Furthermore, the NAS-mask and NAS-boundary decoders are complementary to each other. On the one hand, the mask segmentation of the cell nucleus requires the boundary to be restricted. On the other hand, the boundary segmentation of the cell nucleus requires the mask as a supplement. Therefore, the collaborative work of the cell nucleus mask and boundary segmentation of the cell nucleus is the key to solving the problem of connected nuclei in overlapping cell nuclei. Based on the above discussions, the two feature maps, separately generated from the NAS-mask and NAS-boundary, are fused to perform the collaborative work of cell nucleus mask and boundary segmentation, thereby enhancing the sensitivity of cell nucleus boundaries.

### 4.2 NAS framework

One of the other reasons why our proposed Bloss-DDNet is effective is attributed to the application of the NAS framework (Elsken et al., 2019; Shaw et al., 2019). Unlike other segmentation networks, this study introduces the NAS strategy in segmentation tasks to separately determine the dual-task decoding branches' 1^#^ and 2^#^ decoder modules.

Specifically, there are significant differences in semantic information between the mask and the boundary of the cell nucleus. Therefore, based on the above reasons, it is necessary to determine the corresponding decoding for the mask and boundary of the cell nucleus separately to parse the different semantic information corresponding to the decoding feature map generated from the same shared encoder. However, determining which kinds of convolution to use, the connection method between convolutions, and selecting the hyperparameters of the 1# and 2# decoder modules is a technical issue to achieve optimal segmentation of overlapping cell nuclei. When facing the above technical problems, inspired by the NAS strategy in the classification network (Elsken et al., 2019), we introduce this NAS strategy to the overlapping cell nucleus segmentation of cervical clinical LBC images. It is precisely because the optimal types of convolution kernels to use, the connection method between convolutions, and hyperparameters of the 1^#^ and 2^#^ decoder modules are effectively determined that the segmentation performance of our proposed Bloss-DDNet is further improved.

### 4.3 Bending loss

One of the last reasons our proposed Bloss-DDNet is attributed to the proposed bending loss. The core cause of this problem is the lack of a practical calculation for overlapping boundary loss in the training process, which directly affects the recognition of overlapping boundaries by the subsequently trained network.

Specifically, whether it's binary cross-entropy loss L_*BCE*_, Dice lossL_*DSC*_, or their combination (L_*DSC*_+ L_*BCE*_), they all pay more attention to the mask of cell nucleus segmentation. Therefore, they are adequate for the mask of cell nucleus segmentation, especially when combined (L_*DSC*_+ L_*BCE*_). However, this is also the main reason for the problem of nuclear boundary connectivity. Therefore, based on the above reasons, the proposed bending loss L_*Bend*_ is applied to the NAS-boundary. It is sensitive to the curvature changes of the nuclear boundary in the overlapping cell nucleus, thereby solving the nuclear boundary connectivity problem. Meanwhile, the effective and collaborative work between L_*DSC*_+ L_*BCE*_ and L_*Bend*_ in the training process of the network further enhances the segmentation performance of the cell nucleus segmentation mask, supplementing the boundary of the cell nucleus mask.

### 4.4 The generalization of the overlapping cell nucleus segmentation model based on the proposed Bloss-DDNet

The generalization of the overlapping cell nucleus segmentation models is a core requirement for clinical applications, directly affecting the practicality and value of medical artificial intelligence, and serving as a key bridge connecting technology and clinical practice (Shi et al., 2023; Schreier et al., 2020).

The generalization of the model is directly reflected in the performance on the multi-center LBC dataset. According to the comparative experimental results on the performance of overlapping cell nucleus segmentation models, our proposed overlapping cell nucleus segmentation model based on the proposed Bloss-DDNet exhibits good generalization performance on the public datasets of CNSeg and MoNuSeg. The good generalization performance on the public datasets of CNSeg and MoNuSeg demonstrates that the proposed model can adapt to these changes in cervical clinical LBC images, providing a solid foundation for clinical use of this proposed model for overlapping cell nucleus segmentation.

### 4.5 Limitations

Although we propose a novel bending loss and dual-task decoding network (Bloss-DDNet) for overlapping cell nucleus segmentation of cervical clinical LBC images, our study still has certain limitations. First, the encoder is shared by 1^#^ and 2^#^ decoder modules, and the NAS strategy is only applied to separately determine the 1^#^ and 2^#^ decoder modules of the dual-task decoding branches. Second, we restricted the types and hyperparameters of sub-modules in the NAS framework to include only the dilated convolution, depthwise separable convolution, 2D MaxPool layer, and SE Attention. Third, although the proposed network has made significant progress in overlapping cell nucleus segmentation of cervical clinical LBC images, the hyperparameters of the proposed network still require further optimization. Lastly, the most representative clinical cases also require further evaluation in practical settings.

### 4.6 Perspectives and future work

Based on the limitations of this study, we outline the perspective and future work. First, the two decoder modules, along with their respective decoder modules for dual-task decoding branches, can be attempted based on the NAS strategy to achieve better segmentation of the overlapping cell nucleus in cervical clinical LBC images. Second, other types and hyperparameters of sub-modules in the NAS framework can also be added to determine more optimized 1^#^ and 2^#^ decoder modules of the dual-task decoding branches.

## 5 Conclusion

This study proposed a novel Bloss-DDNet for overlapping cell nucleus segmentation of cervical clinical LBC images. Specifically, dual-task decoding branches composed of a shared encoder module and dual-task optimal decoder modules, determined by the NAS framework, are designed to decode the mask and boundary. Additionally, two feature maps, separately generated from the dual-task decoding branches, are fused to enhance the sensitivity to cell nucleus boundaries. In addition, a bending loss, which focuses on the curvature variation characteristics of the intersection of overlapping cell nucleus boundaries, is introduced to the loss function to constrain the training process of the dual-task decoding branch and increase the constraint on the cell nucleus boundary. The results show that all evaluation metrics of the proposed Bloss-DDNet achieved the best performance on the public data sets. Therefore, the proposed Bloss-DDNet can effectively solve the segmentation problem of overlapping cell clusters and nuclei in clinical LBC images, providing strong support for subsequent clinical auxiliary diagnosis of cervical cancer.

## Data Availability

Publicly available datasets were analyzed in this study. This data can be found here: https://github.com/jingzhaohlj/AL-Net.
